# The Adjuvant Double Mutant *Escherichia coli* Heat Labile Toxin Enhances IL-17A Production in Human T Cells Specific for Bacterial Vaccine Antigens

**DOI:** 10.1371/journal.pone.0051718

**Published:** 2012-12-20

**Authors:** Susannah Leach, John D. Clements, Joanna Kaim, Anna Lundgren

**Affiliations:** 1 Gothenburg University Vaccine Research Institute (GUVAX), Dept. of Microbiology and Immunology, University of Gothenburg, Gothenburg, Sweden; 2 Dept. of Microbiology and Immunology, Tulane University School of Medicine, New Orleans, Louisiana, United States of America; National Council of Sciences (CONICET), Argentina

## Abstract

The strong adjuvant activity and low enterotoxicity of the novel mucosal adjuvant double mutant *Escherichia coli* heat labile toxin, LT(R192G/L211A) or dmLT, demonstrated in mice, makes this molecule a promising adjuvant candidate. However, little is known about the mechanisms responsible for the adjuvant effect of dmLT or whether dmLT also has an adjuvant function in humans.

We investigated the effect of dmLT on human T cell responses to different bacterial vaccine antigens: the mycobacterial purified protein derivative (PPD) antigen, tested in individuals previously vaccinated with *Bacillus Calmette-Guérin*, the LT binding subunit (LTB), evaluated in subjects immunised with oral inactivated whole cell vaccines against enterotoxigenic *Escherichia coli*, and *Streptococcus pneumoniae* whole cell vaccine antigens, tested in subjects naturally exposed to pneumococci. We found that dmLT enhanced the production of IL-17A by peripheral blood mononuclear cells in response to all antigens tested. dmLT had comparable effects on IL-17A responses to PPD as the single mutant LT(R192G) adjuvant, which has demonstrated clinical adjuvant activity in humans. Neutralisation of IL-1β and IL-23, but not IL-6, suppressed the IL-17A-enhancing effect of dmLT. Furthermore, CD4+ T cells produced higher levels of IL-17A when stimulated with monocytes pulsed with PPD and dmLT compared to PPD alone, supporting an important role of antigen presenting cells in enhancing IL-17A responses. dmLT also potentiated mitogen-induced IL-17A and IL-13 production. However, dmLT had variable influences on IFN-γ responses to the different stimuli tested.

Our demonstration of a potent ability of dmLT to enhance human Th17 type T cell responses to bacterial vaccine antigens encourages further evaluation of the adjuvant function of dmLT in humans.

## Introduction

Mucosal infections remain a major global health problem and a considerable cause of child mortality and morbidity [1]. However, there are still relatively few mucosal vaccines available [2]. One reason for this is the lack of a mucosal adjuvant approved for human use. The ADP-ribosylating bacterial enterotoxin heat-labile toxin (LT) produced by enterotoxigenic *Eschericia coli* (ETEC), or cholera toxin (CT) produced by *Vibrio cholerae*, are very powerful mucosal adjuvants [3]. However, the toxicity of these molecules precludes their use in humans. LT and CT are A-B toxins that bind to the gut epithelial cells, inducing a series of cellular events resulting in the irreversible activation of adenylate cyclase, leading to increased levels of intracellular cAMP. This in turn causes an efflux of chloride ions and concomitant osmotic movement of water across the gut into the lumen, resulting in watery diarrhoea in humans [3]. At the same time, LT and CT have potent adjuvant activity and have been used to enhance immune responses against a large number of different vaccines in preclinical studies [3]. To enable use in humans, several parallel attempts have been made to develop mutated LT toxins with lower enterotoxicity yet retain adjuvanticity, including LT(R192G), or single-mutant LT (mLT) [3]. This molecule has a single mutation within the subtended disulphide region of the A subunit, which prevents trypsin-activation and cleavage of the A-subunit, with subsequent reduced toxicity but retained potent adjuvant activity in animal models [3]. However, when mLT was tested in clinical trials together with oral inactivated *Helicobacter* and *Campylobacter* killed whole-cell vaccines, 15–20% of test subjects experienced diarrhoea [4], [5]. To further detoxify mLT, an additional mutation was introduced in the A2-A1 activation loop site [6]. This toxoid, LT(R192G/L211A), double-mutant LT or dmLT, has been demonstrated to enhance immune responses to whole cell vaccines against ETEC, *Streptococcus pneumoniae* and *Helicobacter pylori* in different mouse models [7], [8].

dmLT and mLT, as well as the native toxins CT and LT, can all enhance both antibody and T cell responses [3], [8], [9]. Although the mode of action by which these toxins and toxin derivatives induce adjuvant effects remains largely unknown, recent studies in murine models have helped to shed light on some mechanisms, including the demonstration of an important role for Th17 cells. For example, mucosal immunisation with irradiated anthrax spores or ovalbumin in combination with CT induces vaccine-specific Th17 cells [10]. The IL-17A produced was required for IgA and IgG1 antibody production, as demonstrated by a significant impairment of mucosal IgA and systemic IgA and IgG1 production after oral immunisation of IL-17A deficient mice. Recently, LT was also shown to promote protective Th17 responses to *Bordetella pertussis* after parenteral administration; innate IL-1β and IL-23 production was found to be central for this Th17 induction [11]. Furthermore, sublingual or buccal administration of a killed whole-cell pneumococcal vaccine together with dmLT has been shown to induce potent IL-17A responses, which were associated with protection against pneumococcal colonisation [8]. Thus, preclinical studies indicate that dmLT, LT and CT all potently enhance Th17 responses.

Considering the potent adjuvant effects of dmLT, LT and CT demonstrated in mice and the recent indications that Th17 responses may be involved in the adjuvant function of these molecules, we investigated the effect of dmLT on human T cell responses *in vitro*, with a particular focus on IL-17A responses. We used mycobacterial purified protein derivative (PPD) as our primary model vaccine antigen, since mycobacteria are known to give rise to both Th1 and Th17 responses in humans [12], [13] and both types of responses have been demonstrated to be important for protection against mycobacteria in studies in mice [14]. We also evaluated the effect of dmLT on T cell responses to ETEC LT binding subunit (LTB) in cells from subjects immunised with whole cell ETEC prototype vaccines, and to pneumococcal whole cell vaccine antigens in cells from subjects naturally exposed to pneumococci. We show that dmLT promotes the production of IL-17A in human T cells in response to all tested antigens. The Th17-potentiating effect of dmLT was at least partly exerted via antigen presenting cells (APCs), involving production of IL-1β and IL-23.

## Materials and Methods

### Volunteers and collection of specimens

Healthy volunteers, previously vaccinated with *Bacillus Calmette-Guérin* (BCG) (n = 26, median 32 years, range 19–58 years, 73% females) were recruited from students and staff at the University of Gothenburg and heparinised venous blood was collected for evaluation of responses to mycobacterial PPD antigen. A subgroup (n = 8, median 31 years, range 23–58, 63% females) of these volunteers was also used for evaluation of responses to a *S. pneumoniae* whole cell vaccine antigen, with the assumption that all adults have been naturally exposed to this organism. Responses to ETEC LTB were analysed in another group of volunteers (n = 20, median 27 years, range 19–46 years, 45% females) participating in a phase I ETEC vaccine trial (OEV-120; EudraCT 2009-015741-23, ISRCTN23764070). In this trial, the safety and immunogenicity of two oral inactivated ETEC vaccines were compared: one whole cell vaccine expressing the ETEC colonisation factor CFA/I administered in combination with recombinant cholera toxin B-subunit (CTB) (n = 10), and one whole cell vaccine recombinantly over-expressing CFA/I given together with the more LT-like toxoid LCTB*A* (n = 10) [15], [16]. LCTB*A* is a hybrid protein in which seven amino acids in the LTB protein have replaced amino acids in the corresponding positions in the CTB molecule [15]. Volunteers that had been vaccinated with the oral cholera vaccine Dukoral®, or travelled to a country where ETEC infections are common within the last 5 years, were excluded from all experiments.

The Ethical Review Board for Human Research of the Gothenburg Region approved the study, and written informed consent was obtained from each volunteer before participation.

### Cell preparation

Peripheral blood mononuclear cells (PBMCs) were immediately separated from the heparinised whole blood by density-gradient centrifugation on Ficoll-Paque (GE Healthcare Bio-Sciences, Sweden). CD4+ T cells were isolated from PBMCs by positive selection with magnetic beads (Dynabeads; Dynal AS, Norway). CD4+ cells from a subset of volunteers were further separated into naive (CD45RA+) T cells using negative depletion of CD45RO+ memory/effector cells with magnetic beads (Miltenyi Biotec GmbH, Germany). CD14+ monocytes were isolated from PBMCs by positive selection using magnetic beads (Miltenyi Biotec GmbH, Germany). All isolated cell fractions contained >95% pure cells, as determined by flow cytometric analysis.

### Antigens, mitogens and toxins/toxin derivatives

Cells were stimulated with combinations of antigens and toxins or toxin derivatives, or medium alone as a control. The antigens used were PPD (Statens Serum Institut, Denmark), LTB (Etvax AB, Sweden) and whole cell pneumococcal vaccine antigen (WCA, kindly provided by R. Malley, Children's Hospital, Harvard Medical School, USA). WCA was derived from strain Rx1AL-, a capsule and autolysin-negative mutant and prepared as previously described [8], [17]. Cells were also stimulated with the mitogen phytohaemagglutinin (PHA, Remel, USA). The toxins/toxin derivatives used were LT, mLT, dmLT, and LTB (J. Clements, Tulane University, USA [6], [18]) as well as CT (Sigma-Aldrich, Germany).

### Cell stimulations

All cells were cultured in DMEM F12 medium (200 µl/well) supplemented with 50 µg/ml gentamicin and 5% human AB+ serum at 37° in 5% CO_2_. PBMCs (1.5×10^5^ per well) were cultured in duplicate or triplicate wells in round-bottomed 96-well plates. Cells were stimulated with PPD (5 µg/ml), WCA (1 µg/ml) or PHA (1 µg/ml) and increasing concentrations (0.1, 1 and 10 µg/ml) of toxin/toxin derivatives, or with LTB (10 µg/ml) together with 1 µg/ml dmLT. After 72 hours of mitogen stimulation or 120 hours of antigen stimulation, supernatants were collected for cytokine analysis by ELISA, and the cell proliferation was measured by incorporation of radioactive thymidine. For neutralisation of cytokines in culture supernatants, anti-cytokine antibodies α-IL-1β (clone 8516), α-IL-6 (clone 1936), α-IL-23 (clone 727753) and isotype control antibodies (IgG1, clone 11711 and IgG2b, clone 20116) were added at the start of the cultures and after 48 hours (5 µg/ml for all, except α-IL-23; 0.5 µg/ml, all from R&D, USA).

To determine whether dmLT can influence T cells directly in the absence of APCs, CD4+ T cells (5×10^4^ cells per well) were stimulated with anti-CD3/28 beads (Dynabeads; Dynal AS, Norway) in a 1∶1 ratio with and without dmLT (1 and 10 µg/ml), cultured for 72 hours, after which supernatants were collected.

To determine if the toxins/toxin derivatives can influence T cells via APCs, CD14+ monocytes (7.5×10^4^ per well) were stimulated with antigen, with and without dmLT, for 24 hours. The adherent monocytes were then washed 3 times with cell culture medium, and CD4+ T cells (7.5×10^4^ per well) were added to the wells and cultured for 120 hours, when supernatants were collected and cytokine responses were analysed.

To assess the effect of soluble factors in culture supernatants on the cytokine production, PBMCs (1.5×10^5^ cells per well) were cultured with PPD (5 µg/ml) or dmLT (1 µg/ml), or a combination of PPD and dmLT for 48 hours and supernatants were then collected. To remove residual dmLT, supernatants were incubated on microtitre ELISA plates coated with GM1 ganglioside (3 nmol/ml). 100 µl supernatant was incubated at 37°C on a series of 6 ELISA plates, 30 minutes on the first plate and 10 minutes on each of the 5 consecutive plates. The successful depletion of dmLT was confirmed by ELISA, as previously described [19]; the remaining concentration of dmLT was ≤0.001 µg/ml. These supernatants were then added to PBMCs (1.5×10^5^ cells per well) and cultured for 120 hours. Alternatively, the supernatants were added to CD4+CD45RA+ naïve T cells (4×10^4^ cells per well) stimulated with anti-CD3/28 beads (1∶1 ratio) and cultured for 120–240 hours, after which the cells were restimulated with phorbol myristate acetate and ionomycin for 6 hours. Supernatants were then collected and cytokine responses were analysed.

### Analysis of cytokines

All supernatants were stored at −70°C until cytokine analysis. The concentrations of IL-17A and IFN-γ (eBioscience, USA) and IL-13 (R&D, USA) were determined by ELISA.

### Flow cytometry

For flow cytometric analysis, the cells were stained with the following antibodies: anti-CD19 FITC, anti-CD3 PE, anti-CD14 PerCP, anti-CD4 PerCP and anti-CD3 APC (BD, San José, USA). All cells were fixed in formaldehyde and analysed on a FACSCalibur Flow Cytometer (BD, San José, USA) equipped with a blue and red laser, and results analysed with the FlowJo software (Tree Star Inc, USA).

### Proliferation analysis

The cell proliferation was determined by pulsing the cells with 0.5 µCi of [^3^H]thymidine/well (Amersham, Arlington Heights, USA) for 8 hours. The incorporation of radioactivity was measured with a scintillation counter.

### Statistical analysis

The Friedman test with Dunn's multiple comparison post test and the Wilcoxon signed rank test were used for statistical comparisons, as indicated. P<0.05 was considered statistically significant.

## Results

### dmLT enhances production of IL-17A in PBMCs in response to PPD

**Figure 1:**
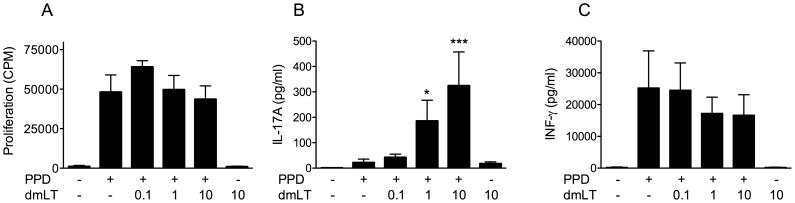
dmLT enhances IL-17A responses to PPD in BCG immunised individuals. PBMCs from BCG vaccinated volunteers (n = 9) were stimulated with PPD in combination with increasing concentrations of dmLT (0, 0.1, 1 and 10 µg/ml). Proliferation (A), and concentration of IL-17A (B) and IFN-γ (C) in culture supernatants were determined. Responses to medium alone and dmLT alone (10 µg/ml) are shown by the leftmost and rightmost bars in each graph. Bars represent mean + SEM. Results shown are from 6 independent experiments. Statistical analysis was performed using the Friedman test with Dunn's multiple comparison post test. * P<0.05 and *** P<0.001; compared to cells stimulated with PPD alone.

To determine if dmLT influences human T cell responses, PBMCs were stimulated with dmLT in combination with vaccine antigens or mitogens, and the production of Th1 and Th17 type cytokines and cell proliferation were analysed. Considering the importance of both Th1 and Th17 cells in immunity to mycobacteria [12], [13], [14], we chose PPD as our primary model vaccine antigen, and analysed responses to this antigen in PBMCs collected from individuals previously immunised with the BCG vaccine. PPD stimulation alone induced strong proliferative responses in all individuals tested (Fig. 1A), demonstrating the presence of memory/effector T cells specific for this recall antigen, while stimulation with dmLT alone did not induce proliferation at any concentration tested (0.1–1 µg/ml; data not shown, 10 µg/ml; see Fig. 1A). Stimulation of cells with a combination of PPD and increasing concentrations of dmLT resulted in comparable proliferation as stimulation with PPD alone (Fig. 1A). Cells stimulated with PPD alone produced IL-17A, although at relatively low levels (Fig. 1B). Cells stimulated with 0.1–1 µg/ml dmLT alone responded with undetectable levels of IL-17A in the majority of volunteers (data not shown), while stimulation with 10 µg/ml dmLT gave rise to low, but detectable IL-17A production (Fig. 1B). However, significantly higher production of IL-17A was induced when PPD and dmLT were added in combination to the cell cultures. The IL-17A production tended to increase already when 0.1 µg/ml dmLT was added compared to PPD alone (2-fold mean increase) and addition of 1 µg/ml and 10 µg/ml resulted in significantly elevated IL-17A production compared to stimulation with PPD alone (9-fold and 15-fold mean rises, respectively). Stimulation with PPD alone also induced production of IFN-γ, while dmLT alone did not stimulate any detectable IFN-γ production (Fig. 1C). In contrast to IL-17A responses, there were no major differences in IFN-γ production when increasing concentrations of dmLT were added to the cultures.

Taken together, these results show that dmLT enhances the production of the Th17 cytokine IL-17A in response to PPD in PBMCs from BCG vaccinated volunteers, but that dmLT has little effect on the production of the Th1 cytokine IFN-γ in response to this antigen.

### dmLT enhances IL-17A and IL-13 responses to the mitogen PHA

**Figure 2:**
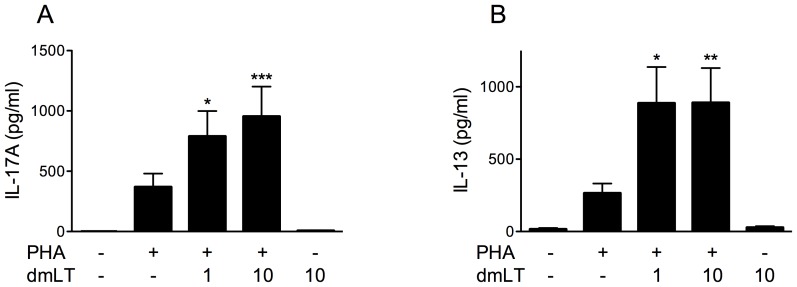
dmLT enhances IL-17A and IL-13 responses to PHA stimulation. PBMCs from volunteers (n = 9) were stimulated with PHA, in combination with increasing concentrations of dmLT (0, 1 and 10 µg/ml) and production of IL-17A (A) and IL-13 (B) were determined. Responses to medium alone and dmLT alone (10 µg/ml) are shown by the leftmost and rightmost bars in each graph. Bars represent mean + SEM. Results shown are from 6 independent experiments. Statistical analysis was performed using the Friedman test with Dunn's multiple comparison post test. * P<0.05, ** P<0.01 and *** P<0.001; compared to cells stimulated with PHA alone.

To analyse the influence of dmLT on Th2 responses, which cannot be detected after stimulation with PPD, PBMCs were also stimulated with dmLT in combination with the mitogen PHA. PHA stimulation induced production of high levels of not only IL-17A (Fig. 2A) and IFN-γ (data not shown), but also the Th2 associated cytokine IL-13 (Fig. 2B). IL-13 was chosen as a model Th2 cytokine in these analyses, since this cytokine is normally produced at higher levels by human T cells than other Th2 cytokines and can easily be detected in culture supernatants after stimulation. Addition of dmLT in combination with PHA induced increased levels of IL-17A (Fig. 2A), although the difference between IL-17A responses in cells stimulated with PHA alone and PHA plus 1 or 10 µg/ml dmLT was only 3- and 4-fold, respectively. Similar to the IL-17A responses, IL-13 production also increased when 1 µg/ml dmLT was added to the cultures (mean 4-fold increase) but the IL-13 response did not increase further when 10 µg/ml dmLT was added (Fig. 2B). IFN-γ responses to PHA were not significantly influenced by addition of dmLT at any concentration tested (data not shown). Thus, our results show that dmLT can promote Th17 and Th2 type responses to PHA stimulation.

### mLT, LT and CT also enhance IL-17A responses to PPD

To determine if other ADP-ribosylating toxins, detoxified mutants or subunits related to dmLT also potentiate IL-17A responses, mLT, LT, LTB and CT were tested in our *in vitro* assay using PPD as the model antigen. mLT, having only one of the two amino acid substitutions introduced in dmLT, enhanced IL-17A responses to a similar extent as dmLT at both 1 and 10 µg/ml (Table 1). Addition of LT resulted in stronger IL-17A responses than addition of either mLT or dmLT, giving rise to 3-fold more IL-17A at 1 µg/ml (P>0.05) and 4-fold more IL-17A at 10 µg/ml (P<0.01) compared to dmLT. CT gave rise to comparable (P>0.05) high levels of IL-17A production as LT at 1 µg/ml in most volunteers, but lower production than LT and comparable levels as dmLT at 10 µg/ml in a majority of subjects. LTB did not influence the IL-17A production at any concentration tested. In line with the results obtained with dmLT, LT, mLT, LTB and CT had no effect on IFN-γ production at any concentration tested (P>0.05, data not shown).

**Table 1:** IL-17A production in response to PPD and increasing concentrations (1 and 10 µg/ml) of ADP-ribosylating toxins, detoxified mutants or subunits.

	PPD	PPD + 1 µg/ml	PPD + 10 µg/ml
**dmLT**	23 (0–126)^a^	162 (16–637)	350 (47–1188)**
Fold rise^b^		7.2	15.5
**mLT**	23 (0–126)	206 (37–595)	288 (23–602)**
Fold rise		9.2	12.8
**LT**	23 (0–126)	764 (194–1973)	907 (332–2509)**
Fold rise		33.9	40.3
**CT**	23 (0–126)	289 (44–2423)*	275 (0–941)
Fold rise		12.8	12.2
**LTB**	23 (0–126)	12 (0–72)	30.5 (0–204)
Fold rise		0.5	1.4

a IL-17A concentration (pg/ml) in supernatants from cells isolated from BCG vaccinated volunteers (n = 6) expressed as medians (range). Results shown are from 3 independent experiments.

b Fold rises in IL-17A concentration as compared to stimulation with PPD alone.

* P<0.05 and **P<0.01; compared to cells stimulated with PPD alone.

These results thus demonstrate that different toxins and toxoids related to dmLT which include the enzymatic A-subunit also enhance human IL-17A responses *in vitro*. dmLT has a comparable effect as mLT, whereas native LT and CT have even stronger potentiating effects than dmLT at low concentrations.

### dmLT enhances IL-17A production in CD4+ T cells

**Figure 3:**
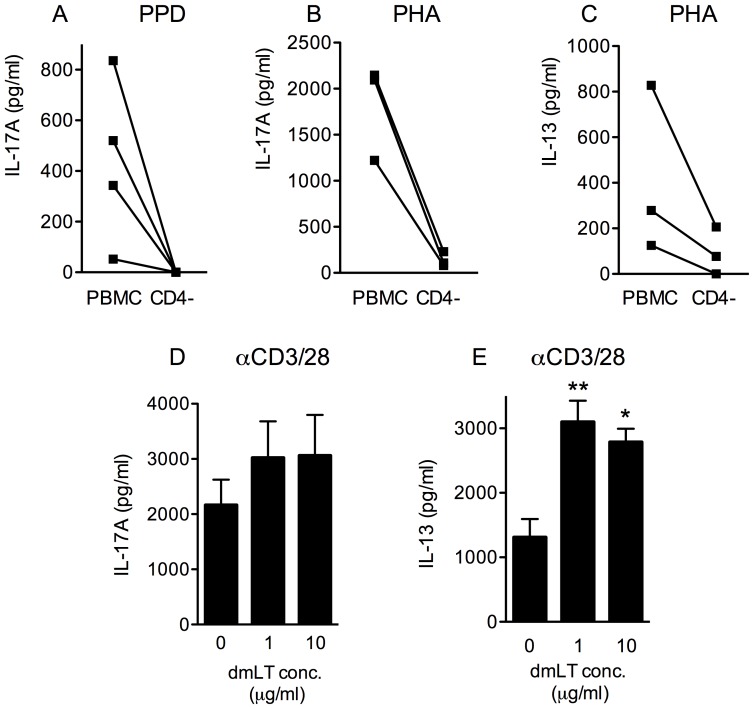
dmLT enhances IL-17A and IL-13 responses in CD4+ T cells. PBMCs and PBMCs depleted of CD4+ T cells isolated from BCG vaccinated volunteers were stimulated with PPD (A, n = 4), or PHA (B–C, n = 3), in combination with 10 µg/ml dmLT, and the IL-17A production was determined. (D–E) CD4+ T cells from another set of volunteers (n = 6) were stimulated with beads coated with anti-CD3/CD28 antibodies and increasing concentrations (0, 1 and 10 µg/ml) of dmLT, and the IL-17A (D) and IL-13 (E) concentration in culture supernatants were determined. Bars represent mean + SEM. Results shown are from 2 (A–C) or 3 (D–E) independent experiments. Statistical analysis was performed using the Friedman test with Dunn's multiple comparison post test. * P<0.05 and ** P<0.01; compared to cells stimulated with anti-CD3/CD28 beads alone.

CD4+ T cells are the main producers of IL-17A, although CD8+ T cells, γδ T cells and NKT cells have also been reported to produce IL-17A [20]. To determine if the strong IL-17A responses detected in cultures stimulated with PPD and dmLT originated from CD4+ T cells, IL-17A responses were compared in PBMCs and PBMCs depleted of CD4+ T cells. In cell cultures lacking CD4+ T cells, the IL-17A production induced by stimulation with PPD and 10 µg/ml dmLT was reduced to non-detectable levels in all tested individuals (Fig. 3A). A similar dependency on CD4+ T cells was also detected after stimulation with PHA, both for IL-17A (Fig. 3B) and IL-13 (Fig. 3C).

Next, we wanted to determine if the enhanced cytokine production induced by dmLT was dependent on APCs, or if the CD4+ T cells may also respond to dmLT directly. Purified CD4+ T cells were therefore stimulated with beads coated with anti-CD3/CD28 antibodies, with or without dmLT, in the absence of any other cells. Addition of 1 µg/ml dmLT caused a small, non-significant increase in IL-17A production (1.5-fold increase), and the production did not increase further when 10 µg/ml dmLT was added (Fig. 3D). However, the IL-13 production increased significantly (P<0.01) when CD4+ T cells were directly stimulated with 1 µg/ml dmLT (mean 2-fold increase, Fig. 3E), yet did not increase further when 10 µg/ml dmLT was added (P<0.05 compared to anti-CD3/CD28 beads alone). Thus, the strong dose-response seen when increasing concentrations of dmLT were added to PBMCs stimulated with PHA was not seen in CD4+ T cells stimulated in the absence of APCs. To verify that this difference was not due to the different types of polyclonal stimulation (anti-CD3/CD28 versus PHA), PBMCs were also stimulated with anti-CD3/CD28 antibodies in combination with dmLT. This resulted in a similar pattern of increased IL-17A production in response to increasing dmLT concentrations as in PHA-stimulated cultures (data not shown), further verifying the importance of APCs for enhancing the IL-17A responses.

These results thus indicate that dmLT can influence IL-13 production by CD4+ T cells directly, but does not appear to directly affect IL-17A production. Thus, APCs are likely to be able to further enhance the IL-17A production in response to dmLT.

### dmLT can enhance IL-17A production from T cells via soluble factors and monocytes

**Figure 4:**
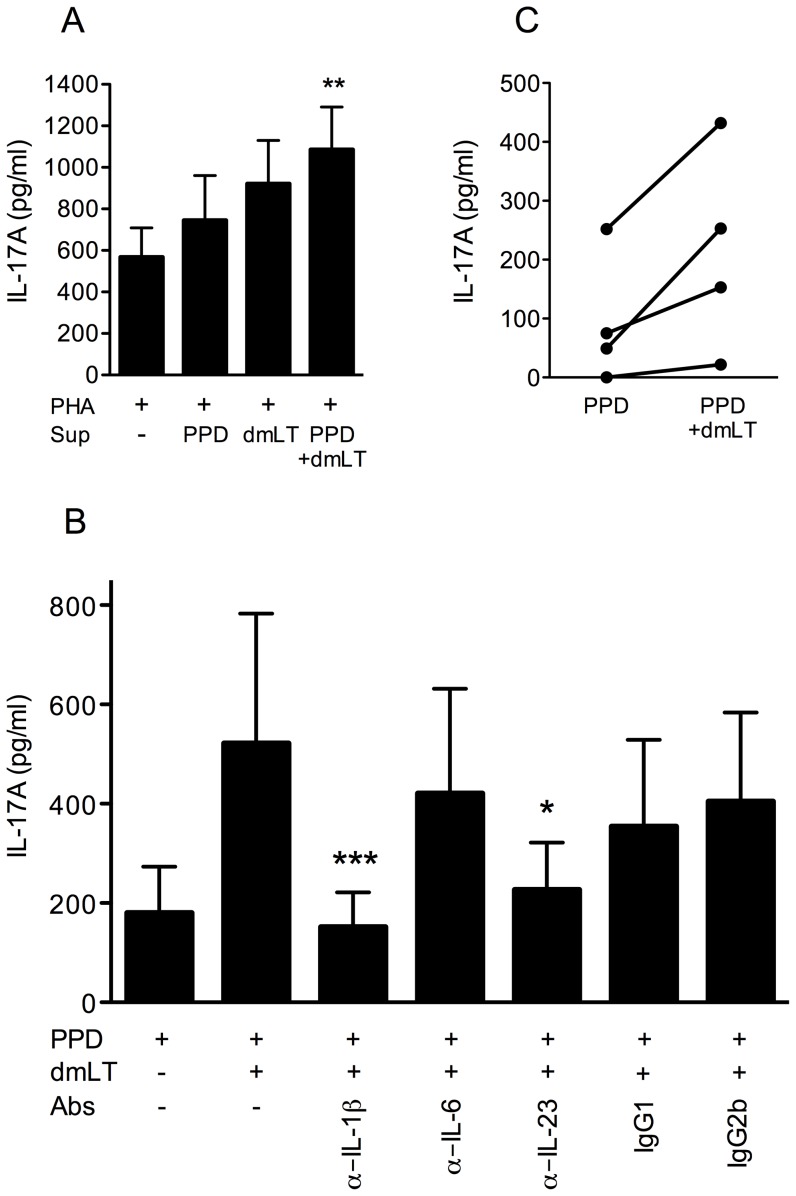
dmLT enhances IL-17A production from T cells via soluble factors and monocytes. (A) PBMCs from BCG vaccinated volunteers (n = 7) were stimulated with PHA in the presence of supernatants (Sup) derived from PBMCs stimulated with PPD, dmLT or PPD + dmLT, and the IL-17A production was determined. Results shown are from 3 independent experiments. (B) PBMCs from BCG vaccinated volunteers (n = 6) were stimulated with PPD and dmLT in the presence and absence of neutralising antibodies (Abs) against IL-1β, IL-6 and IL-23, and the IL-17A production was determined. Results shown are from 3 independent experiments. (A and B) Bars represent mean + SEM. Statistical analysis was performed using the Friedman test with Dunn's multiple comparison post test. * P<0.05, ** P<0.01 and *** P<0.001; compared to cells stimulated with PHA alone (A) or PPD plus dmLT (B). In (B), indicated differences were also significant (P<0.05) compared to treatments with isotype control antibodies. (C) CD14+ monocytes isolated from BCG vaccinated volunteers (n = 4) were pulsed with PPD alone or together with 10 µg/ml dmLT, and then washed. CD4+ T cells were then added to the monocytes, and the resulting IL-17A production was determined. Results shown are from 2 independent experiments.

To determine if soluble factors such as cytokines produced by APCs may be involved in enhancing IL-17A responses after stimulation with dmLT, we stimulated PBMCs polyclonally with PHA in the presence and absence of supernatants derived from PBMCs stimulated with PPD, dmLT or a combination of both stimuli (Fig. 4A). To avoid potential effects of any dmLT remaining in these supernatants, dmLT was depleted by incubating the supernatants in GM1-coated plates, before adding the supernatants to the new cultures. The remaining concentration of dmLT in these supernatants was ≤0.001 µg/ml; this concentration had no effect on cytokine production from PBMCs either alone or in combination with PPD in control experiments (data not shown). These supernatants, which were collected after 48 h, contained no detectable levels of IL-17A. We found that supernatants derived from PBMCs stimulated with PPD plus dmLT significantly increased IL-17A production when compared to cells stimulated with PHA alone in the absence of any added supernatant. Supernatants collected from cells stimulated with dmLT alone also tended to increase the IL-17A production. In contrast, supernatants derived from cells stimulated with PPD alone did not significantly influence the IL-17A production. Addition of extra PPD (5 µg/ml) to cells stimulated with PHA did not enhance the IL-17A production (data not shown), verifying that the enhanced IL-17A production was not a result of PPD remaining in the supernatant. The results were verified in repeat experiments using supernatants derived from PBMCs from another volunteer (data not shown).

Since responses to PHA were evaluated as early as after 72 hours in these cultures, the effects detected were most likely primarily a result of the influence of dmLT on memory/effector T cells, rather than on naive T cells, which produce little cytokines after short-term stimulation ([21], [22] and A. Lundgren, unpublished data). To evaluate if polarisation of naive T cells into IL-17A producing effector cells may also be influenced by soluble factors, CD4+CD45RA+ T cells, which are highly enriched for naive cells, were isolated and stimulated with beads coated with anti-CD3/CD28 antibodies in the presence of the same supernatants as described above. However, no effect on IL-17A production by the naive T cells were observed after addition of any of the supernatants, either by directly measuring the levels of IL-17A in culture supernatants after 5–10 days of stimulation, or after restimulating the cells with PMA (data not shown). These results suggest that the soluble factors induced by stimulation with dmLT obtained in our PBMC culture system may have stronger effects on memory/effector T cells than on naive T cells, although this needs to be verified in more extensive studies.

We hypothesised that the cytokines IL-1β, IL-6 and IL-23 may be involved in enhancing IL-17A production from memory/effector T cells after stimulation by dmLT, since these cytokines are primarily produced by APCs, and are known to be important for induction and sustainment of IL-17A production [23]. To test this hypothesis, IL-1β, IL-6 and IL-23 were neutralised by addition of monoclonal antibodies in PBMC cultures stimulated with dmLT in combination with PPD. Addition of anti-IL-1β or anti-IL-23 antibodies reduced the IL-17A production to levels comparable to those detected in cultures stimulated with PPD alone (Fig. 4B). In contrast, anti-IL-6 antibodies or isotype control antibodies had no significant impact on the IL-17A levels.

Since APCs are a dominant source of IL-1β and IL-23 [24], [25], we wished to establish whether monocytes pulsed with dmLT and PPD could induce increased IL-17A production from CD4+ T cells. Purified CD14+ monocytes were stimulated with PPD alone or in combination with 1 µg/ml dmLT for 24 hours, and the monocytes were then washed thoroughly before CD4+ T cells were added to the cultures, limiting the direct contact between CD4+ cells and dmLT. Analysis of IL-17A levels in supernatants collected 5 days later showed that monocytes pulsed with PPD in the presence of dmLT produced increased levels of IL-17A production in all 4 tested individuals (mean 3-fold increase), compared to stimulation with PPD alone (Fig. 4C).

Collectively, these results demonstrate that dmLT can enhance IL-17A production in CD4+ T cells via effects on monocytes and support an important role for IL-1β and IL-23 in mediating and/or sustaining the effect of dmLT on IL-17A production from CD4+ memory/effector T cells.

### dmLT enhances IL-17A responses to components of novel ETEC and pneumococcal vaccines

To determine if dmLT can enhance IL-17A responses to other bacterial vaccine antigens than PPD, we evaluated the effect of dmLT on responses to the LTB component of candidate ETEC vaccines and to a pneumococcal whole cell vaccine antigen preparation (WCA).

**Figure 5:**
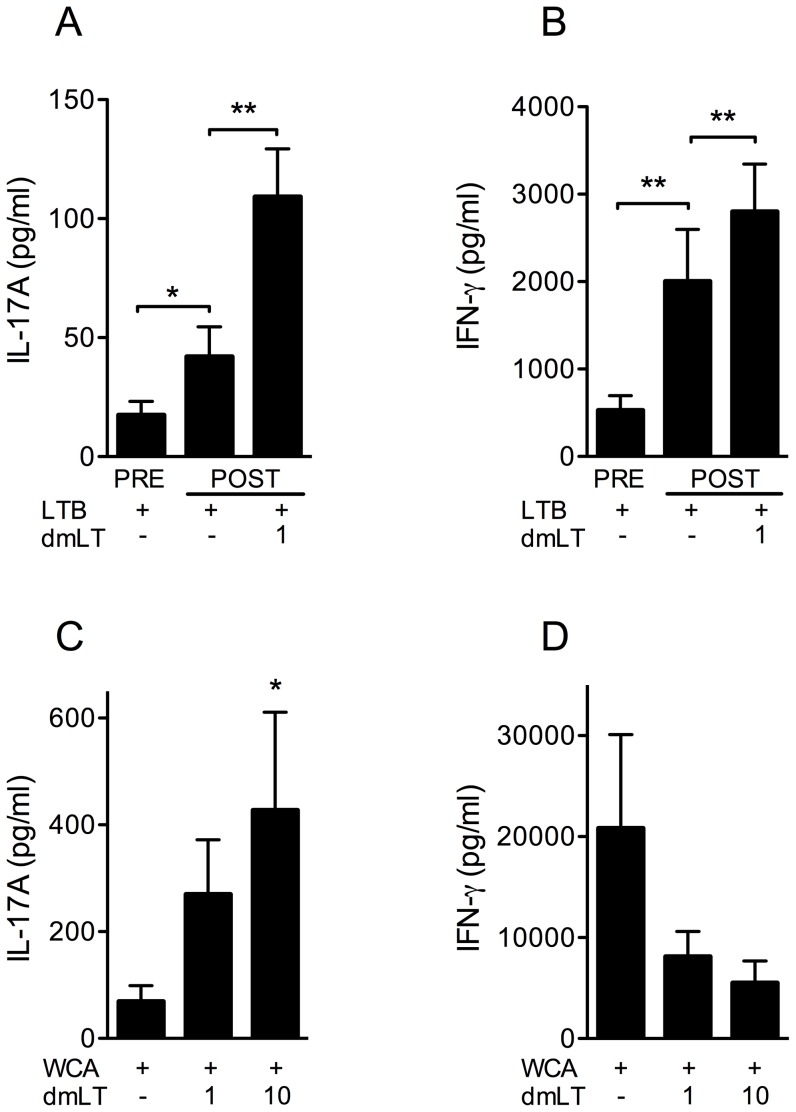
dmLT enhances IL-17A responses to components of novel ETEC and pneumococcal vaccines. (A and B) PBMCs from 20 volunteers collected pre and post-vaccination with oral inactivated whole cell vaccines against ETEC containing CTB or LCTB*A*, were stimulated with 10 µg/ml LTB with and without 1 µg/ml dmLT, and the resulting IL-17A (A) and IFN-γ (B) production was determined. Statistical analysis was performed using the Wilcoxon signed rank test. Results shown are from 6 independent experiments. (C and D) PBMCs from 8 volunteers were stimulated with WCA and increasing concentrations (0, 1 and 10 µg/ml) of dmLT, and the resulting IL-17A (C) and IFN-γ (D) production was determined. Statistical analysis was performed using the Friedman test with Dunn's multiple comparison post test. Results shown are from 6 independent experiments. (A–D) Bars represent mean + SEM. * P<0.05, ** P<0.01; compared to cells stimulated with LTB (A and B) or WCA (C and D) alone. In A and B, separate comparisons of post- versus pre-vaccination responses are also indicated.

Responses to LTB were analysed in PBMCs collected from volunteers immunised with oral inactivated whole cell vaccines against ETEC containing CTB or the CTB/LTB hybrid molecule LCTB*A*. PBMCs were collected before vaccination (day 0), and 1 week after administration of the second vaccine dose (day 21), and were stimulated with LTB (10 µg/ml) alone or in combination with 1 µg/ml dmLT. The LCTB*A* hybrid molecule and CTB differ only in 7 amino acids and immunisation with both molecules gives rise to antibodies that bind both LTB and CTB (unpublished data). Since comparable T cell cytokine and proliferative responses to LTB and dmLT were detected in subjects immunised with vaccines containing CTB and LCTB*A* (data not shown), responses in these two immunisation groups are reported together in figure 5. LTB-stimulation alone gave rise to production of low levels of IL-17A (Fig. 5A) as well as IFN-γ (Fig. 5B) before vaccination, and the responses increased significantly one week after administration of the second vaccine dose in subjects immunised with CTB as well as LCTB*A*, demonstrating that the immunisation gave rise to both Th17 and Th1 type T cell responses. Addition of dmLT increased the production of IL-17A as well as IFN-γ after immunisation and the effect was comparable in the two immunisation groups. A low dose of dmLT was chosen in these experiments (1 µg/ml) to avoid a potential antigen effect of this molecule, since stimulation of PBMCs with 1 µg of LTB only gives rise to marginal T cell responses after ETEC vaccination (data not shown). Addition of 1 µg/ml extra LTB to cultures stimulated with 10 µg/ml LTB did not influence the cytokine production. Furthermore, PBMCs isolated from subjects immunised with either CTB or LCTB*A* responded with low and comparable production of cytokines to stimulation with 1 µg/ml dmLT alone both before and after immunisation (data not shown), supporting that the observed response to dmLT was not an antigen effect, but rather an effect of the adjuvant function of dmLT.

We have recently shown that pneumococcal WCA stimulates IL-17A responses in T cells from healthy adults and children [22], probably as a result of previous natural exposure to *S. pneumoniae*. Consistent with the earlier data, we found that stimulation of PBMCs with WCA alone gave rise to production of IL-17A in most volunteers (Fig. 5C). The IL-17A production was enhanced by the addition of increasing concentrations of dmLT in a similar fashion as previously shown after stimulation with PPD plus dmLT. However, although WCA stimulation also resulted in IFN-γ production, a strong tendency towards suppressed IFN-γ responses was observed in the presence of 1 or 10 µg dmLT (Fig. 5D).

Taken together, these results show that dmLT enhances IL-17A responses to ETEC LTB and pneumococcal WCA, while having variable effects on IFN-γ responses to these two different types of antigen.

## Discussion

dmLT is a promising mucosal adjuvant candidate, but little is currently known about the mechanisms responsible for the adjuvant effect or whether dmLT has an adjuvant function in humans. In this study, we show that dmLT can enhance human IL-17A responses in PBMCs to the model bacterial antigen PPD in BCG immunised individuals, while having little effect on IFN-γ production or proliferation in response to this antigen. Depletion experiments verified that the IL-17A produced by PBMCs in the presence of PPD in combination with dmLT primarily originated from CD4+ T cells. We focused our studies on responses to PPD, since IL-17A has been shown to be produced by T cells specific for mycobacteria in humans and to play an important role in protection against mycobacterial infection in mice [12], [13], [14]. We also analysed effects of dmLT on responses to pneumococcal whole cell vaccine antigen in individuals naturally exposed to *S. pneumoniae*. IL-17A has been shown to be of critical importance for the protection against pneumococcal colonisation in mice immunised with pneumococcal whole cell vaccine or naturally exposed to live pneumococcal bacteria [26], [27], likely via recruitment and/or activation of neutrophils and macrophages [26], [28]. Furthermore, sublingual administration of dmLT together with WCA significantly reduces pneumococcal colonisation and enhances IL-17A production [8]. We found that dmLT could enhance IL-17A responses to pneumococcal WCA in PBMCs. In contrast, IFN-γ responses to this antigen were suppressed, which is noteworthy considering that IFN-γ is not necessary for protection against pneumococcal colonisation in mice immunised with pneumococcal WCA [26].

Recent preclinical studies of an oral killed whole cell ETEC vaccine suggest that dmLT can also enhance mucosal and systemic IgA responses to LTB, as well as to ETEC colonisation factors in mice (J. Holmgren, personal communication). Therefore, we also tested the effect of dmLT in *in vitro* cultures of PBMCs isolated from human volunteers orally immunised with prototype oral killed whole cell ETEC vaccines containing an LTB/CTB toxoid component. We found that dmLT could enhance both IL-17A and IFN-γ responses to ETEC LTB. Mucosal antibodies are known to be the major protective mechanism against ETEC infection, whereas T cells may play a more indirect role, such as promoting antibody responses and memory development. In line with such a role, recent studies suggest that IL-17A can support antibody dependent protection via a variety of mechanisms, including enhancement of B cell recruitment, germinal centre formation and IgA production, as well as increase the secretion of IgA over epithelial cells as a result of enhanced expression of the poly-Ig receptor [29], [30], [31], [32], [33], [34]. Our observations of the ability of dmLT to enhance IFN-γ responses to LTB in individuals immunised with ETEC vaccines, as well as to promote IL-13 production in response to PHA, also suggests that dmLT has the potential to influence antibody production via increased Th1 and/or Th2 responses.

While the *in vivo* adjuvanticity of dmLT remains to be demonstrated in humans, mLT has already been shown to enhance IgA antibody secreting cells as well as IFN-γ responses to a killed whole cell *Campylobacter jejuni* vaccine in a clinical trial [5], [35]. When we compared dmLT and mLT in our *in vitro* assay, we found that these molecules enhanced IL-17A responses to a similar extent. Our findings are consistent with *in vivo* data from mice, showing that dmLT and mLT have a comparable adjuvant function [6], [7]. Our *in vitro* data also show that in most volunteers LT and CT enhanced IL-17A responses in PBMCs even more potently than dmLT or mLT when tested at 1 µg/ml. However, at the highest concentration tested (10 µg/ml), CT induced lower levels of IL-17A in most subjects, while LT, dmLT and mLT efficiently enhanced IL-17A production further, which may suggest a toxic or suppressive effect of CT on PBMCs at this concentration. LTB had no effect on IL-17A responses, which is consistent with recent data suggesting that IL-17 induction by LT derivatives is dependent on the presence of an enzymatically active A-subunit [9]. While both CT and LT are clearly too toxic to be used as adjuvants in humans, mLT has reduced enterotoxicity. However, mLT has been shown to cause diarrhoea in a small proportion of human volunteers given a high dose of mLT alone (100 µg) or a moderate dose of mLT (25 µg) in combination with the *Campylobacter* vaccine, limiting the practical use of this adjuvant in humans [5], [35]. In contrast, dmLT did not show any signs of enterotoxicity when recently tested alone at high oral doses in a clinical study (100 µg) or in preclinical tests (L. Bourgeois, personal communication and [6]). The comparable *in vitro* performance of dmLT and mLT demonstrated here provide further support that dmLT may be successfully used as an adjuvant in humans.

After oral administration *in vivo*, small amounts of dmLT adjuvant may interact directly with T cells, since enterotoxins have been shown to be taken up by M cells and to be transported to the underlying cells in the mucosa, including T cells [3]. However, a major proportion of the *in vivo* adjuvant function of these molecules is likely to be mediated indirectly, via effects on APCs and epithelial cells that in turn may influence T cell activity. We investigated the role of APCs in our culture system by stimulating isolated CD4+ T cells polyclonally in the absence of APCs, and found that dmLT had only a marginal effect on IL-17A production under these conditions, while the enhancement was amplified in the presence of APCs. Furthermore, we demonstrated that monocytes pulsed with antigen in combination with dmLT induced more IL-17A in CD4+ T cells compared to monocytes pulsed with antigen alone, supporting a role for APCs in potentiating IL-17A responses. We also found that secreted factors were sufficient to enhance IL-17 responses, since supernatants collected from PBMCs stimulated with antigen plus dmLT, but depleted of any remaining dmLT, could enhance IL-17A responses when transferred to polyclonally stimulated PBMCs. Furthermore, neutralisation of IL-1β and IL-23, but not IL-6, inhibited the IL-17A potentiating effect of dmLT, showing that the effect of dmLT may be at least partially mediated via these cytokines. Consistent with this notion, IL-1β, and to some extent also IL-23, have been shown to enhance IL-17A production in T cell receptor stimulated memory CD4+ T cells from humans, while IL-6 alone had little effect [36], [37]. IL-23 and IL-1β were also recently shown to be critically involved in driving IL-17A production in response to LT both *in vitro* and *in vivo* in mice [11].

We have focused our *in vitro* studies on the effect of dmLT on memory/effector T cell responses to recall antigens, while *in vivo*, dmLT may enhance both primary and secondary responses. Although extensive controversy has existed regarding the role of different cytokines in induction of the Th17 phenotype in naïve cells, and in mice versus humans, recent studies support a critical role for IL-1β in combination with IL-23, or in some studies IL-6, in both species, whereas TGF-β seems to be dispensable [38], [39], [40]. However, when we activated human naïve CD4+ T cells polyclonally in the presence of supernatants collected from PBMCs stimulated with dmLT with or without antigen, we could not detect any major influence of these supernatants on IL-17A induction in the naïve T cells, although such supernatants strongly enhanced IL-17A production in memory/effector T cells. These findings may be consistent with data suggesting that IL-1β, IL-23 and IL-6 have a more important IL-17A promoting function in memory than in naive T cells, and that TGF-β plus IL-21 may be the most Th17 polarising cytokine combination for naïve T cells [37]. However, the influence of dmLT on naive T cell responses in humans merit further studies, including experiments using different APCs and types of stimuli.

In our *in vitro* study, dmLT had variable effect on IFN-γ responses to different stimuli, having little effect on IFN-γ responses to PPD and PHA, while enhancing IFN-γ responses to LTB and suppressing IFN-γ responses to the pneumococcal vaccine antigens. LT has been shown to suppress production of the Th1 inducing cytokine IL-12 by dendritic cells *in vitro* [11], [41], but *in vivo*, LT can promote IFN-γ as well as IL-4 and IL-10 production, although to a lesser extent than IL-17A [11]. Whether dmLT influences IL-12 production is presently unclear, and requires further investigation. It is likely that antigens of different purity and complexity, as those tested in our study, may induce different signals from accessory cells that influence the IFN-γ production. Additional microenvironmental signals may further modify the responses *in vivo*, potentially resulting in enhancement of both Th17 and Th1 responses.

Taken together, the current data from our study as well as previous studies in mice, suggest that dmLT, LT and CT may all influence T cells via soluble factors secreted from APCs. Further studies are needed to fully elucidate the mechanisms involved for each combination of antigen and enterotoxin/toxoid, to explain differences observed between memory and naive cells and between *in vitro* and *in vivo* findings. We have recently initiated a phase I trial where dmLT is tested together with a more definite formulation of the inactivated whole cell ETEC vaccine and analyses of Th17, as well as Th1 and Th2 type T cell responses, will be important parts of this study. This trial is likely to increase our understanding of how dmLT may influence primary T cell responses and will also give an opportunity to analyse the potential correlation between Th17 responses and antibody production.

In conclusion, we have demonstrated that dmLT enhances the production of IL-17A from human CD4+ effector/memory T cells isolated from volunteers immunised with BCG and ETEC vaccines, as well as from subjects naturally exposed to *S. pneumoniae*. We also showed that the enhancing effect on PPD responses is dependent on production of IL-1β and IL-23. dmLT also enhanced IFN-γ responses to LTB in individuals immunised with ETEC vaccines. Our results highlight the importance of careful examination of T cell responses in coming clinical vaccine trials in order to further clarify how the dmLT adjuvant may promote different components of human immune responses.
